# Enhancing the anti-tumour activity of ^177^Lu-DOTA-octreotate radionuclide therapy in somatostatin receptor-2 expressing tumour models by targeting PARP

**DOI:** 10.1038/s41598-020-67199-9

**Published:** 2020-06-23

**Authors:** Carleen Cullinane, Kelly Waldeck, Laura Kirby, Buck E. Rogers, Peter Eu, Richard W. Tothill, Rodney J. Hicks

**Affiliations:** 1 Division of Cancer Research, Peter MacCallum Cancer Centre, Melbourne, Victoria Australia; 2 Department of Cancer Imaging, Peter MacCallum Cancer Centre, Melbourne, Victoria Australia; 3 Sir Peter MacCallum Department of Oncology, University of Melbourne, Parkville, Victoria Australia; 4 Department of Clinical Pathology and Centre for Cancer Research, University of Melbourne, Parkville, Victoria Australia; 5 Department of Radiation Oncology, Washington University School of Medicine, St Louis, MO 63110 USA; 6 School of Medicine, Deakin University, Geelong, Victoria Australia

**Keywords:** Cancer models, Cancer therapy, Neuroendocrine cancer

## Abstract

Peptide receptor radionuclide therapy (PRRT) is an important treatment option for patients with somatostatin receptor-2 (SSTR2)-expressing neuroendocrine tumour (NET) though tumour regression occurs in only a minority of patients. Therefore, novel PRRT regimens with improved therapeutic activity are needed. Radiation induced DNA damage repair is an attractive therapeutic target to increase PRRT efficacy and consequently, we have characterised a panel of preclinical models for their SSTR2 expression, *in vivo* growth properties and response to ^177^Lu-DOTA-octreotate (LuTate) PRRT to identify models with features suitable for evaluating novel therapeutic combinations*. In vitro* studies using the SSTR2 expressing AR42J model demonstrate that the combination of LuTate and the small molecule Poly(ADP-ribose) polymerase-1 (PARP) inhibitor, talazoparib led to increased DNA double strand breaks, as assessed by γ-H2AX foci formation, as compared to LuTate alone. Furthermore, using the AR42J tumour model *in vivo* we demonstrate that the combination of LuTate and talazoparib significantly improved the anti-tumour efficacy of LuTate alone. These findings support the clinical evaluation of the combination of LuTate and PARP inhibition in SSTR2-expressing NET.

## Introduction

Neuroendocrine tumours (NET) represent a rare and heterogeneous group of tumours. Characteristically slow growing, these tumours are often diagnosed late in the disease course with locally-advanced or metastatic tumours. A subgroup has associated hypersecretion of hormones that can cause significant morbidity. NET arises from neuroendocrine cells of the diffuse endocrine system with the highest incidence observed in the gastrointestinal tract (70%) and lung (30%)^1,2^. Classification of NET is based on the cell-type, organ of origin or associated hormone secretion, while grading is based on the cell differentiation and, in particular, on proliferation rate^3^. Clinical management is highly complex and may involve surgery, chemotherapy and molecularly targeted therapies. More recently peptide receptor radionuclide therapy (PRRT) has become well established as a key therapeutic modality for NET, particularly those involving the gastrointestinal tract^4^.

Neuroendocrine cells are regulated by hormones acting via G-protein coupled receptors such as the somatostatin receptor (SSTR2). Since many NET share high level expression of SSTR2, this feature has been exploited for diagnosis, staging and the therapeutic targeting of NET^5^. The ^111^In-labelled somatostatin analogue, octreotide and the more recently developed ^68^Ga-labelled analogues have been used widely for diagnostic imaging of SSTR2-expressing tumours by SPECT and PET, respectively^6,7^. Incorporation of other therapeutic radionuclides such as ^177^Lu and ^90^Y, optimisation of the chelating agent and replacement of octreotide with the higher affinity analogue, octreotate have facilitated the development of PRRT as an effective therapy for NET. PRRT is associated with prolonged survival, improved symptom control and quality of life in patients with advanced NET but objective regression of disease is observed in only a minority of patients^8–11^.

The development of novel PRRT regimens with improved therapeutic activity is therefore needed to further enhance the long-term outcomes for patients with NET. However, due to the inherent problems of performing prospective randomised controlled trials in NET patients, identifying such treatment regimens has been challenging and controversial^12^. The addition of radiosensitising chemotherapy to PRRT has been explored in the clinical setting where the combination of 5-fluorouracil (5-FU) and PRRT using ^111^In-octreotate and ^177^Lu-DOTA-octreotate (LuTate) was shown to be safe and well tolerated in patients with progressive NET^13,14^. PRRT in combination with the 5-FU prodrug, capecitabine alone or in combination with temozolomide has also been demonstrated as safe and efficacious in advanced NET^15–18^. More recently, we have demonstrated that patients selected on the basis of uncontrolled symptoms or progression within 12 months on conventional therapy can achieve high objective response rates and prolonged progression-free survival following peptide chemoradionuclide therapy^19^. These results were also recapitulated in a group of patients with a more aggressive phenotype based on ^18^F-fluorodeoxyglucose positron emission tomography (FDG-PET)^20^. These findings encourage the evaluation of other combination therapies with PRRT to further enhance its radiobiological effects.

Poly(ADP-ribose) polymerase-1 (PARP-1) is a 116 kDa protein that plays an important role in the recognition and repair of DNA damage via the base excision repair (BER) pathway. PARP-1 binds to single-strand DNA breaks where it recruits BER proteins to the damage site to execute the repair program^21^. Inhibition of PARP has been an attractive target and successful for therapeutic intervention in cancer^22^. Indeed, preclinical studies have demonstrated that PARP inhibitors sensitise tumour cells to chemotherapy agents such as methylating agents and topoisomerase I inhibitors^23–26^ and external beam radiotherapy^23,27,28^. In the setting of beta emitting radionuclides, which predominantly induce single strand DNA breaks, inhibition of the repair of these lesions via PARP inhibition may lead to conversion of single strand breaks to cytotoxic double strand DNA breaks upon DNA replication. Indeed, preclinical anti-tumour activity of PARP inhibitors has been observed in combination with chemotherapy and beta particle emitting targeted radiotherapy agents^29,30^ and more recently, PARP inhibitors have been shown to enhance the formation and persistence of cytotoxic double strand DNA breaks and potentiate the cytotoxicity of LuTate *in vitro*^31,32^.

In this study we sought to test the hypothesis that inhibition of PARP would potentiate the *in vivo* efficacy of LuTate PRRT. Given the paucity of tractable *in vivo* models of SSTR2-expressing NET has limited the development of novel therapeutic approaches in this setting, the aims of the study were therefore to characterise a panel of cell lines with neuroendocrine features to identify models appropriate for evaluating the anti-tumour activity of combination regimens incorporating SSTR2-targeted PRRT and then employ the model *in vivo* to evaluate the efficacy of the PARP inhibitor, talazoparib in combination with LuTate PRRT.

## Results

### Characterisation of cell line models for SSTR2 expression

**Figure 1:**
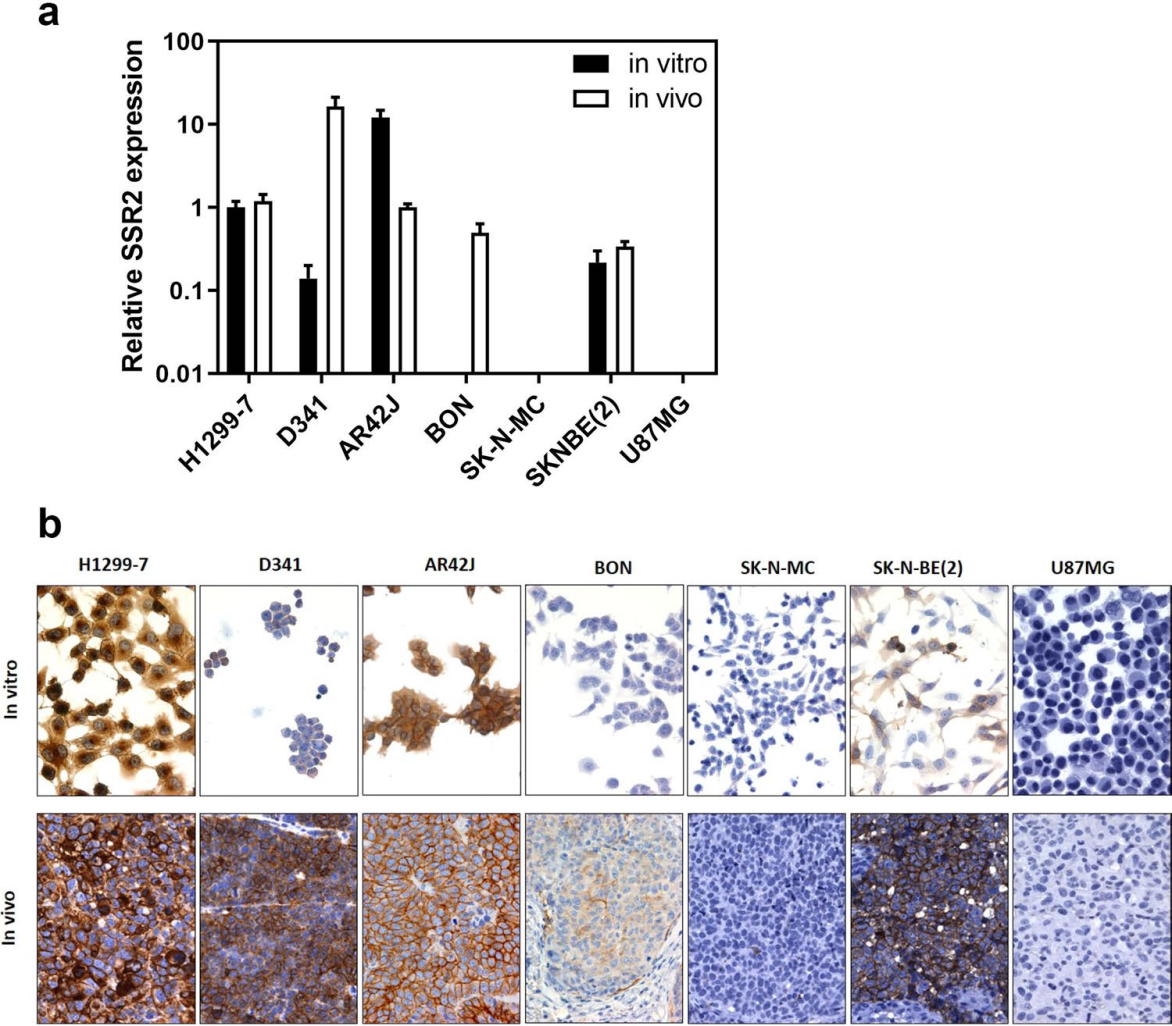
SSTR2 expression across the cell line panel. (**a**) SSTR2 mRNA expression was evaluated using RT-PCR in cells grown in culture and as tumours *in vivo*. Data is normalised to expression in the SSTR2-transfected H1299-7 model *in vitro*. Results represent mean ± SEM of at least three independent values. Where no bar is seen, expression was >100-fold lower than in the H1299-7 reference sample. (**b**) Representative images of cultured tumour cells (upper panels) or xenograft tissue sections (lower panels) stained for SSTR2 expression.

A panel of tumour cell lines with neuroendocrine features comprising a rat exocrine pancreatic tumour (AR42J)^33^, human functioning pancreatic carcinoid (BON)^34^, human medulloblastoma (D341)^35^, human glioma (U87MG)^36^, two human neuroblastomas (SK-N-MC^37^, SK-N-BE(2)^38^) and an SSTR2 transfected human non-small cell lung cancer line (H1299-7)^39^ was initially examined for *in vitro* and *in vivo* expression of SSTR2 mRNA. *In vitro*, wide variation in SSTR2 expression was observed with maximal expression in the AR42J cells, which was 12-fold higher than that in the reference SSTR2 transfected H1299-7 cells (Fig. 1a). D341 and SK-N-BE(2) cells expressed the receptor mRNA at 7- and 5-fold lower levels, respectively, while in the remaining lines (BON, U87MG and SK-N-MC), SSTR2 expression was negligible. In contrast, with the exception of the SK-N-MC and U87MG models, all lines demonstrated robust SSTR2 mRNA expression when grown *in vivo*.

Immunocytochemistry was performed to assess SSTR2 protein expression *in vitro*. The H1299-7, AR42J and SK-N-BE(2) lines exhibited predominantly cytoplasmic staining of SSTR2 while low or no staining was evident in D341, BON, SK-N-MC or U87MG cells (Fig. 1b, upper panels). In contrast, when the cells were grown as tumour xenografts *in vivo*, SSTR2 expression was primarily localised to the cell membrane (Fig. 1b, lower). The most robust membrane staining was observed in the three tumour models (H1299-7, AR42J and SK-N-BE(2)), which had also exhibited the highest cytoplasmic staining when grown *in vitro*. Consistent with the SSTR2 mRNA findings, the D341 and BON models demonstrated markedly higher receptor staining when grown *in vivo* than *in vitro* but no SSTR2 staining was detected in the U87MG or SK-N-MC tumours.

### Characterisation of tumour SSTR2 imaging phenotype

**Figure 2:**
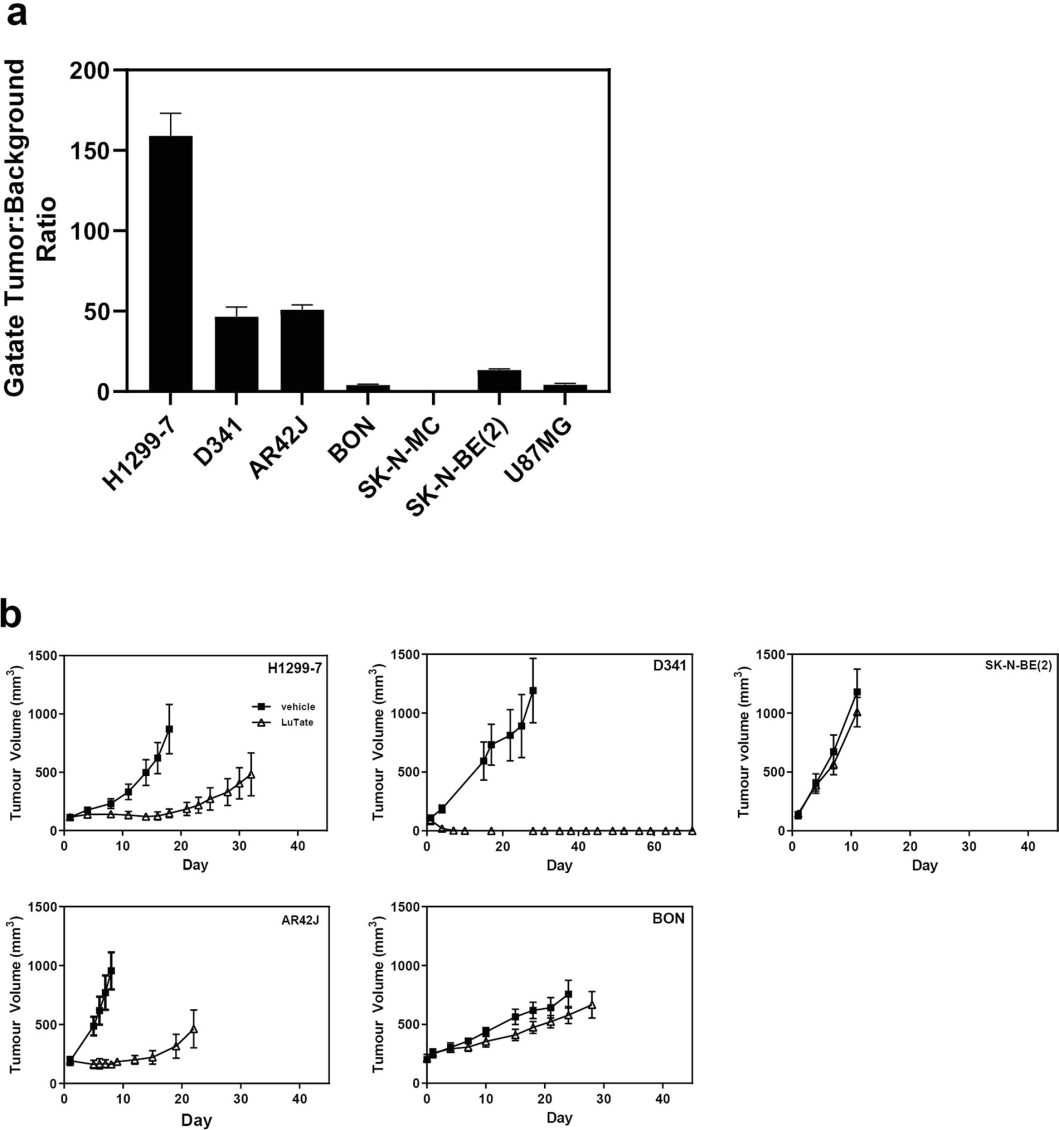
*In vivo* GaTate PET imaging phenotype and LuTate response across the tumour panel. (**a**) Mice bearing subcutaneous tumours were imaged using a small animal PET scanner following administration of GaTate (Images shown in Supp. Figure 1). PET tracer tumour to background uptake ratios were determined and are expressed as mean ± SEM of at least three independent tumours. (**b**) Mice bearing tumours were treated intravenously with saline or 20 MBq LuTate on day 1. Tumour volumes are expressed as mean ± SEM of 4–8 animals/group.

The tumour models were evaluated for SSTR2 expression *in vivo* by ^68^Ga-DOTA-octreotate (GaTate) PET imaging (Fig. 2a, Supplementary Fig. S1). The PET images showed very high tracer binding in the SSTR2 transfected H1299-7 model with a tumour to background binding ratio (TBR) of 159 ± 14 as determined by semiquantitative analysis. High GaTate binding in D341 (TBR = 47 ± 6) and AR42J (TBR = 51 ± 3) tumours was observed while in the SK-N-BE(2) model the TBR was 4-fold lower (TBR = 13 ± 4). U87MG, BON and SK-N-MC tumours demonstrated very low GaTate avidity. Together, these GaTate imaging findings are consistent with the SSTR2 mRNA and protein expression observed *in vivo*.

### Tumour response to LuTate therapy

Cell lines that expressed SSTR2 *in vivo* were implanted into nude mice and once the tumours became well-established the animals were injected intravenously with 20 MBq LuTate and the tumour response evaluated. As seen in Fig. 2b, most tumour models showed similar robust growth kinetics but their response to LuTate varied widely. LuTate treatment of the H1299-7 and AR42J models induced tumour stasis for sixteen and twelve days post dosing, respectively, after which tumour growth rapidly resumed. In contrast, the D341 model, which showed similar SSTR2 expression and GaTate uptake to that of the AR42J model, was highly sensitive to LuTate with complete tumour regression observed for 65 days. The SK-N-BE(2) and BON tumour models which demonstrated low SSTR2 expression and GaTate binding were highly resistant to LuTate treatment.

### Enhancement of DNA damage *in vitro* by combining LuTate with a PARP inhibitor

**Figure 3:**
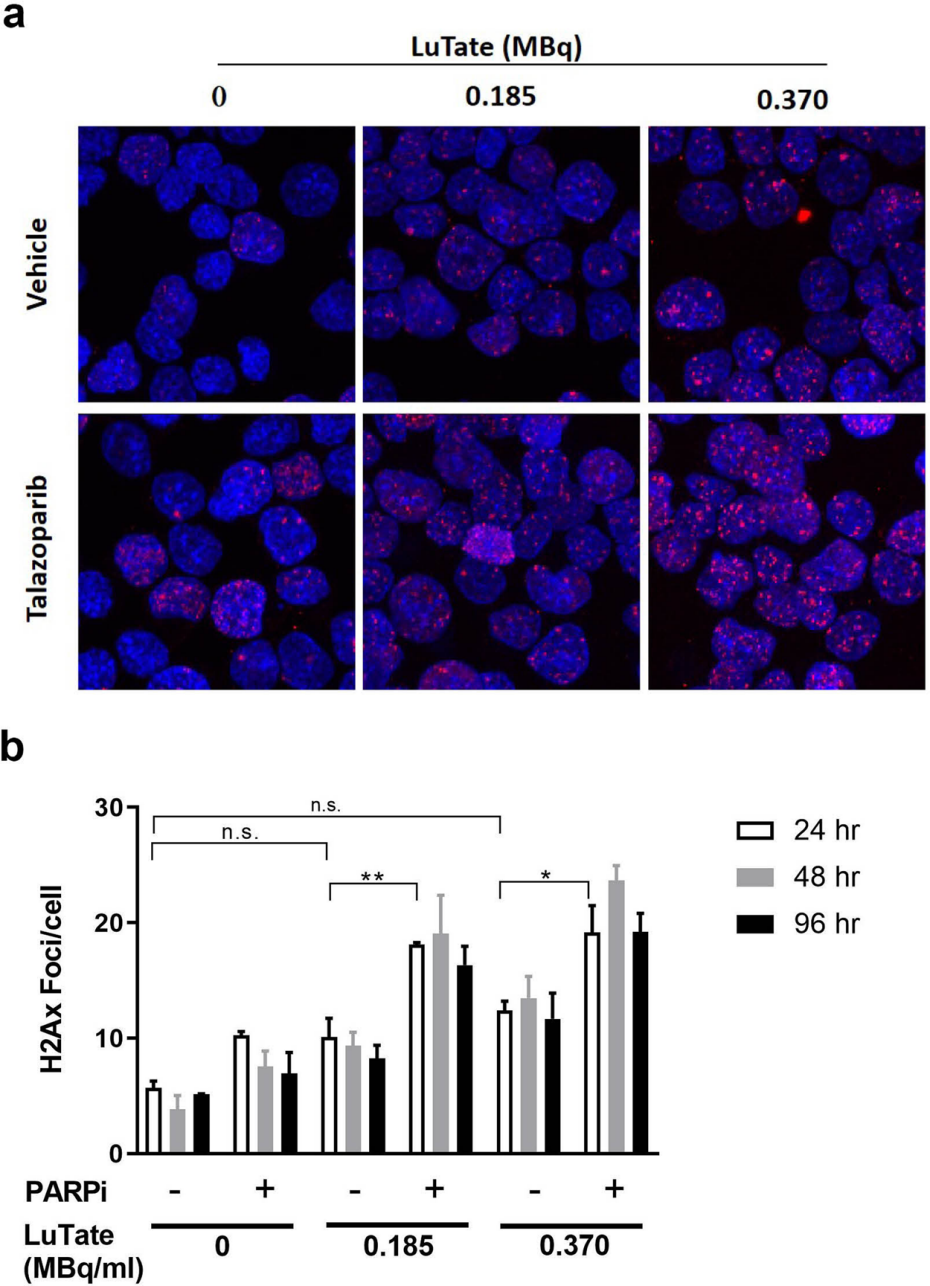
Talazoparib enhances DSB induced by LuTate. AR42J cells were treated with single agents or in combination and assessed for ɣ-H2AX foci formation over 96 hr. (**a**) Representative images of cells stained for ɣ-H2AX are shown. Red, ɣ-H2AX; Blue, DAPI nuclear staining. (**b**) Five images per sample (minimum of 50 cells) were analysed and the average ɣ-H2AX foci per cell quantitated and expressed as mean ± SEM; n = 3; n.s. not significant, *P < 0.05, **P < 0.01.

On the basis of its robust tumour growth properties, *in vivo* SSTR2 expression and response to LuTate PRRT, the AR42J line was then used to explore the ability of a PARP inhibitor to potentiate the effects of LuTate treatment *in vitro* and *in vivo*. AR42J cells were treated with LuTate for two hours before being washed in PBS and assessed for the presence of DNA double strand breaks (DSB) by ɣ-H2AX foci staining (Fig. 3a). At 24 hr after incubation with 185 kBq/ml and 370 kBq/ml LuTate, ɣ-H2AX foci increased modestly from a baseline level of 5.7 ± 0.6 to 10.1 ± 1.6 (*P* = n.s.) and 12.4 ± 0.8 (*P* = n.s.) foci/cell, respectively with these lesion levels persisting over four days (Fig. 3b). The addition of the PARPi, talazoparib, significantly increased DSB levels induced by 185 kBq/ml LuTate to 18.1 ± 0.2 foci/cell (*P* = 0.003) and 370 kBq/ml LuTate to 19.2 ± 2.3 foci/cell (*P* = 0.011) at 24 h with lesions persisting to 96 h.

### *In vivo* efficacy of LuTate PRRT in combination with talazoparib

**Figure 4:**
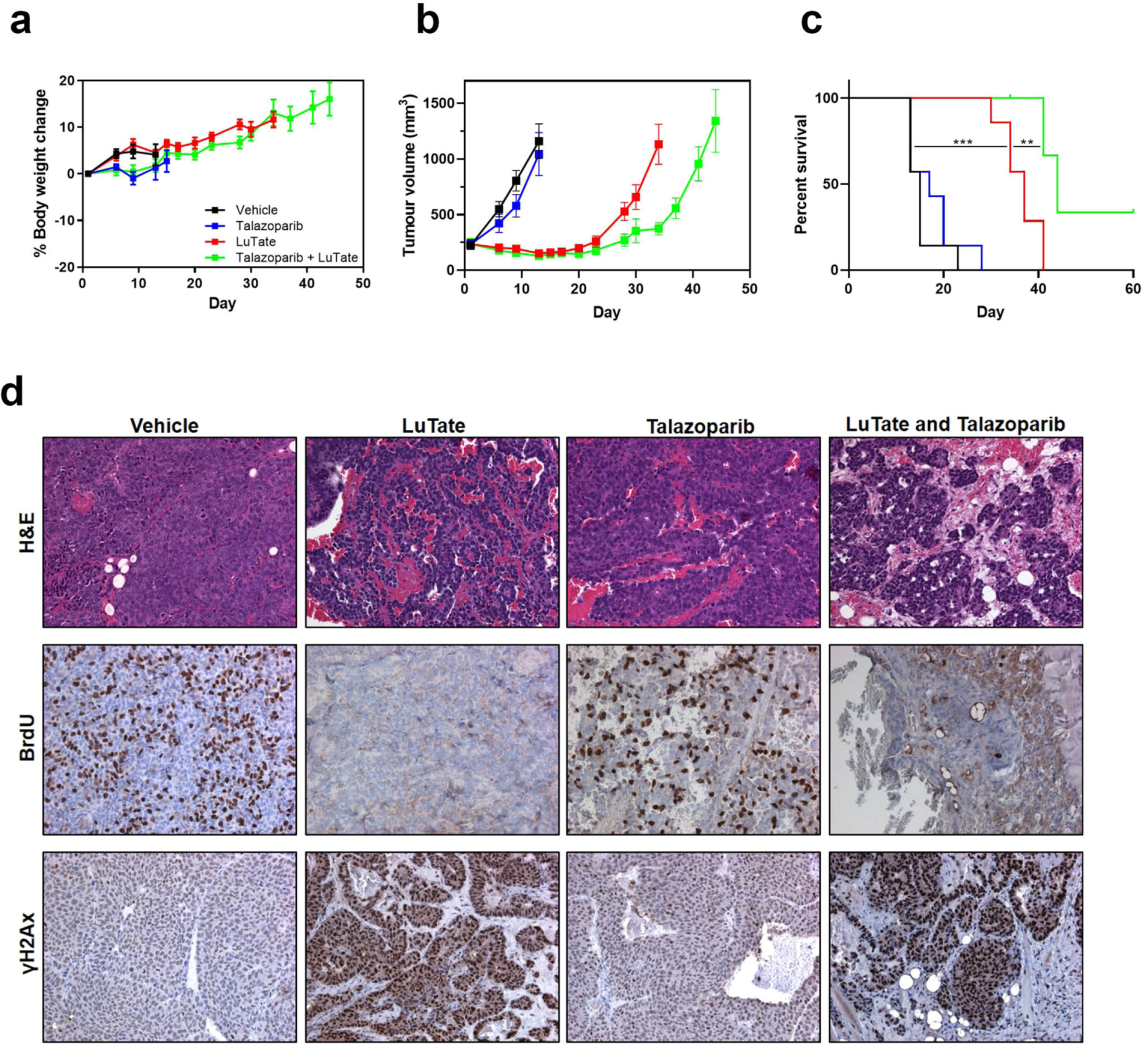
Talazoparib potentiates the anti-tumour activity of LuTate therapy *in vivo*. Mice bearing AR42J tumours were treated with 30 MBq LuTate on day 1 alone or in combination with 0.25 mg/kg talazoparib twice daily on days 1–5. (**a**) Animal body weights were monitored and are expressed as the mean percent change in body weight from day 1 ±SEM. Tumours were measured twice weekly and are presented as (**b**) mean tumour volume ±SEM (n = 7 animals/group) or (**c**) Kaplan Meier survival curves where survival endpoint was defined as the time the tumour volume reached 1200 mm^3^ (right). Tumour volumes are shown until the first mouse was removed from the group due to reaching maximal ethical tumour volume. **P < 0.01, ***P < 0.001. (**d**) Representative haematoxylin and eosin (H&E) staining and BrdU and ɣ-H2AX immunostaining of AR42J tumours harvested at 72 hr following treatment with LuTate and talazoparib.

We next investigated the anti-tumour effects of LuTate in combination with talazoparib in the AR42J xenograft model *in vivo*. Tumour bearing mice (tumour volume 90–460 mm^3^) received a single administration of 30 MBq LuTate, 0.25 mg/kg talazoparib twice daily for 5 days, or the two agents in combination with talazoparib starting on the day of PRRT administration and given over 5 days. The dosing schedule was well tolerated as assessed by the absence of any body weight loss (Fig. 4a) and the impact of treatment on tumour growth is summarised in Fig. 4b. No single agent activity of talazoparib was observed while LuTate treatment resulted in tumour regression for two weeks post therapy (35% regression on day 13), after which the tumours began to regrow. Although the extent of tumour regression after the combination therapy was similar to that for LuTate, the combination treatment significantly prolonged inhibition of AR42J tumour growth (*P* = 0.0028 unpaired *t*-test on day 34). LuTate therapy alone induced a survival benefit (*P* = 0.0002) compared to vehicle while the combination of talazoparib and LuTate further enhanced median survival from 37 days in the LuTate alone group to 44 days (*P* = 0.0025) (Fig. 4c).

To investigate the pharmacodynamics of the combined treatments, AR42J tumours were harvested and analysed at 72 hr post treatment. LuTate treatment alone and in combination with talazoparib induced marked changes in tumour morphology consistent with the induction of necrosis on haematoxylin and eosin straining while cell proliferation was also reduced to negligible levels as assessed by bromodeoxyuridine incorporation (Fig. 4d). ɣ-H2AX immunohistochemistry, performed as a biomarker of DNA DSBs, showed low levels background staining in talazoparib treated tumours and high levels of positive staining in both the LuTate and combination treated tumours.

## Discussion

A recent randomised controlled trial demonstrated the efficacy of LuTate therapy in patients with advanced midgut NET^10^. However, as complete responses to LuTate are rare, novel approaches are needed to further potentiate its clinical activity. Preclinical efficacy studies are of great value for evaluating novel treatments but the lack of robust and well validated preclinical models of SSTR2-expressing NET available for the assessment of novel combination regimens incorporating SSTR2 targeted PRRT is well documented^40–42^. In this study, we therefore characterised a panel of cell lines with neuroendocrine features to identify a line suitable for such studies. Using such a model, we then demonstrated the preclinical efficacy of combining LuTate with the PARPi, talazoparib.

The therapeutic activity of LuTate requires tumour expression of its target receptor, SSTR2. Our data demonstrate that the expression and cellular localisation of SSTR2 differed depending on the context in which cells were grown, with high membrane localised SSTR2 expression seen in tumour xenografts while lower, predominantly cytoplasmic SSTR2 expression seen in cells cultured *in vitro*. The basis of this discordance is unclear but likely reflects the influence of different microenvironmental factors such as cell-cell interactions and the availability of cognate ligand on receptor expression and localisation. The ability of cells to modulate their SSTR2 expression has also been reported in response to treatment with a range of pharmacological agents and radiation^43–45^, highlighting the potential of exploiting modulation of receptor expression to increase the therapeutic efficacy of SSTR2 targeted radionuclide therapy. The low expression and restricted membrane localisation of SSTR2 *in vitro* observed in our study, however, highlights an important limitation of *in vitro* models for the appropriate assessment of the cellular effects of LuTate.

Our results show that while SSTR2 expression is necessary for response to LuTate therapy *in vivo*, it is not sufficient. This is exemplified by the D341 and AR42J tumour models which demonstrated similar GaTate-avidity but highly disparate responses to LuTate (Fig. 2b). Tumour response to LuTate is likely to reflect differences in cell intrinsic factors such as integrity of DNA damage response (DDR) and cell survival pathways together with environmental factors such as tumour hypoxia. The D341 cell line harbours a deleterious mutation in the tuberous sclerosis gene, *TSC2*, a key regulator of the mTOR signalling pathway^46^. A case report of severe radiosensitivity in a patient with tuberous sclerosis, a disease characterised by loss of TSC2 function^47^, is indeed consistent with the potent response of the D341 cell line to LuTate PRRT. As inactivating mutations in the mTOR pathway have been reported in pancreatic NET^48^, further studies are needed to investigate the potential of *TSC2* inactivating mutations as a biomarker for selection of patients for PRRT. Mutations in DNA damage repair pathway genes, including *BRCA2* and *CHEK2* have also been reported in pancreatic NET^48^ and may promote tumour sensitivity to LuTate, as reported in BRCA2 mutant prostate cancer treated with Lu-177 PSMA therapy^49^.

Using a robust tumour model endogenously expressing high levels of SSTR2, we demonstrated for the first time the *in vivo* efficacy of LuTate PRRT in combination with a PARP inhibitor. These results are in agreement with, and extend the findings of, two recent reports which describe the *in vitro* radiosensitisation of LuTate PRRT by PARP inhibitors. Using an osteosarcoma cell line transfected to overexpress SSTR2, Nonnekens *et al*.^31^ showed olaparib potentiated the *in vitro* cytotoxicity of LuTate while Purohit *et al*.^32^ also showed PARPi mediated potentiation of LuTate cytotoxicity in 2D monlayer and 3D spheroid NET cultures. Furthermore, while a number of *in vivo* studies have demonstrated the enhanced efficacy of external beam radiotherapy when given in combination with PARPi^27,50^, the only reported study combining a PARPi with radionuclide therapy showed that a ^177^Lu-labelled anti-EGFR antibody given in combination with rucaparib was more effective against a breast tumour model than either agent alone^30^.

PRRT holds great promise for the treatment of NET and our studies using a well characterised SSTR2-expressing preclinical tumour model demonstrate that the anti-tumour activity of LuTate PRRT can be enhanced by the PARPi, talazoparib. These preclinical findings provide a strong rationale for the clinical evaluation of PARP inhibitors in combination with PRRT in SSTR2-expressing NET.

## Materials and Methods

### Cell lines, drugs and peptide receptor radionuclide therapy

SSTR2 transfected A427 cells (A427–7) were grown as previously described^39^. Upon STR profiling analysis (Australian Genome Research Facility, Melbourne, Australia) this cell line matched (>90%) to another non-small cell lung cancer cell line, H1299 and thus is now referred to as H1299-7. BON cells^51^ were kindly provided by Dr H. Timmer-Bosscha, Department of Medical Oncology, University Medical Center Groningen, The Netherlands. SK-N-BE(2) cells were from DSMZ (Germany) and SK-N-MC, U87MG, AR42J and D341 cells were from ATCC. ^177^LuCl_3_ was purchased from IDB, Holland and [DOTA^0^,Tyr^3^]octreotate was from Advanced Accelerator Applications SA, France. LuTate (specific activity of 70 MBq/nmol) was prepared as described previously^52^. Talazoparib was purchased from Euroasian Chemicals and solubilised in 10% dimethylacetamide, 5% Solutol HS 15 and 85% PBS for *in vivo* studies and DMSO for *in vitro* studies.

### RT-PCR

RNA was prepared from cell lines grown *in vitro* and from xenografts using the High Pure RNA Isolation kit (Roche). Following cDNA synthesis, quantitative real-time PCR reactions were performed in triplicate for each of three individual samples of each cell line/tumour using the SYBR Green (Applied Biosystems) detection method in a StepOne PCR machine (Applied Biosystems). Human SSTR2 and GAPDH primers have been previously described^39^ and rat SSTR2 and GAPDH primers were designed using the NCBI Primer-BLAST design tool [http://www.ncbi.nlm.nih.gov/tools/primer-blast/; SSTR2 Fw (ACA CCC GGC TTT TCT AGA GC), SSTR2 Rv (TTA CAT AGC GGG CAA GCA CA), GAPDH Fw (CCA GCC CAG CAA GGA TAC TG) and GAPDH Rv (GGT ATT CGA GAG AAG GGA GGG C)] The amplification protocol used was 95 °C for 15 min, followed by 40 cycles of 95 °C for 10 sec, 60 °C for 30 sec. Average Ct values for each of the samples were calculated, relative to GAPDH expression, and relative expression determined using the 2^deltaCT method. [Relative expression = 2^-(deltaCT) where delta CT = (CT target gene − CT housekeeping gene) – negative control CT]^53^. Resulting expression changes were normalised relative to the expression in H1299-7 cells.

### SSTR2 immunocytochemisty

Cells cytospun onto glass slides (D341) or grown on glass chamber slides (all other lines) were fixed in methanol before being stained for expression of SSTR2 (ab134152, Abcam, 1:250) for 1 hr at RT. Cells were then incubated with anti-rabbit Envision+ HRP secondary antibody (Dako, Australia) for 1 hr at RT and staining visualised with DAB chromogen reagent (Dako, Australia). Cells were then counter-stained with heamotoxylin, dehydrated through alcohol and coverslipped. Images were captured using a BX61 microscope (Olympus) at 40x magnification.

### *In vitro* DNA damage assays

AR42J cells were treated for 2 hr with 50 nM talazoparib alone or up to 0.37 MBq/mL LuTate, with and without 50 nM talazoparib. Cells were then washed and further incubated in fresh medium with and without talazoparib, for up to 96 hr. Cells were collected fixed in paraformaldehyde before being cytospun onto glass slides. Cells were incubated in gamma H2AX primary antibody (ab22551, Abcam, 1:500) followed by anti-mouse AlexaFluor 555 secondary Ab (A21424 Life Technologies, 1:500). Slides were mounted in Vectashield +DAPI mounting media (Vector Laboratories) and Z-stacked images were captured on a Nikon scanning confocal microscope at 40x magnification. Automated foci counting was performed using in-house software JQuantPlus, and results shown as the mean foci per cell +/− SEM of three independent experiments.

### Xenograft experiments

All mouse experiments were approved by the Peter MacCallum Cancer Centre Animal Ethics Committee and performed in accordance with the Australian code for the care and use of animals for scientific purposes, 8th Edition, 2013. Balb/c nude mice (Animal Resources Centre, Western Australia) were implanted subcutaneously with 1 mm^3^ tumour pieces (D341) or 5–10 × 10^6^ tumour cells in 50% Matrigel (all other lines). Tumour growth was monitored using digital callipers and volume (mm^3^) calculated by the modified ellipsoid formula; length/2 × width^2^, where length represents the longest longitudinal diameter and width the longest perpendicular diameter^54^. Mice were humanely euthanized once the tumour volume exceeded 1200 mm^3^. LuTate (0.4 µg peptide/20 MBq; 0.6 µg peptide/30 MBq) was diluted in saline and given intravenously in a final volume of 100 µl while talazoparib was given orally at 0.25 mg/kg in a volume of 50 mL/kg twice daily for five days.

For biomarker studies AR42J bearing mice were treated as above and euthanized at 72 hr post LuTate treatment. Bromodeoxyuridine (100 mg/kg) was administered by ip injection one hour prior to harvest. Tumours were excised and fixed in formalin before being embedded in paraffin.

### PET imaging studies

GaTate was prepared as described previously^55^. Tumour bearing mice were injected intravenously with 14.8 MBq GaTate. One hour later the mice were anaesthetised in 2% isoflurane in 50% oxygen in air and placed on the bed of a Philips Mosaic small animal PET scanner and imaged over 10 minutes. Image reconstruction and quantitation was as described previously^56^. Briefly, using the on-board imaging software, a region of interest (ROI) was placed around the entire tumour and a representative background region (to represent non-tumour tissue uptake). Maximum and average pixel values within each ROI were then determined. GaTate uptake was calculated as maximum pixel value within a tumour divided by the average pixel value of the background ROI.

### Immunohistochemistry

Four micron sections of formalin-fixed paraffin-embedded tumours were incubated at 60 °C for 1 hr before being dewaxed. Antigen retrieval (AR) for SSTR2 was performed in a pressure cooker at 125 °C for 3 min, for γ-H2AX, in 10 mM sodium citrate (pH 6.0) and for bromodeoxyuridine (BrdU) in High pH AR buffer (Dako, Australia). Prior to primary antibody incubation slides were treated with 3% H_2_O_2_ and immunostaining was then performed as for immunocytochemistry using appropriate secondary antibodies (Mouse or Rabbit Envision+ HRP secondaries, DAKO, Australia). Primary antibodies used were γ-H2AX (ab22551, Abcam, 1:1000), BrdU (347580, BD Biosciences, 1:200) and SSTR2 (ab134152, Abcam, 1:250). Images were captured using an Olympus BX-61 microscope at 20x or 40x magnification.

### Data analysis

The percentage tumour growth inhibition was determined according to the following formula: 100 × (1−ΔT/ΔC) where ΔC and ΔT were calculated by subtracting the mean tumour volume in the vehicle (C) and treated (T) group on day 1 of treatment from the mean tumour volume in that group on the day of analysis. *In vivo* survival differences were determined using the Mantel-Cox log-rank test in Graph Pad Prism 8.0 (Graph Pad La Jolla, CA) where survival was defined as the time for a tumour volume to reach 1200 mm^3^. Other statistical analyses were conducted using the one-way ANOVA followed by Dunnett’s post hoc test or unpaired *t*-test.

## Supplementary information

Supplementary information.

## References

[1] Bodei L, Cwikla JB, Kidd M, Modlin IM (2017). The role of peptide receptor radionuclide therapy in advanced/metastatic thoracic neuroendocrine tumors. Journal of thoracic disease.

[2] Modlin IM (2008). Gastroenteropancreatic neuroendocrine tumours. The Lancet. Oncology.

[3] Klimstra DS (2010). Pathology reporting of neuroendocrine tumors: application of the Delphic consensus process to the development of a minimum pathology data set. Am J Surg Pathol.

[4] Kwekkeboom DJ (2010). Peptide receptor radionuclide therapy in patients with gastroenteropancreatic neuroendocrine tumors. Semin Nucl Med.

[5] Hicks RJ (2010). Use of molecular targeted agents for the diagnosis, staging and therapy of neuroendocrine malignancy. Cancer imaging: the official publication of the International Cancer Imaging Society.

[6] Krenning EP (1989). Localisation of endocrine-related tumours with radioiodinated analogue of somatostatin. Lancet.

[7] Maecke HR, Hofmann M, Haberkorn U (2005). (68)Ga-labeled peptides in tumor imaging. J Nucl Med.

[8] Villard L (2012). Cohort study of somatostatin-based radiopeptide therapy with [(90)Y-DOTA]-TOC versus [(90)Y-DOTA]-TOC plus [(177)Lu-DOTA]-TOC in neuroendocrine cancers. J Clin Oncol.

[9] Kwekkeboom DJ (2010). Somatostatin-receptor-based imaging and therapy of gastroenteropancreatic neuroendocrine tumors. Endocrine-related cancer.

[10] Strosberg J (2017). Phase 3 Trial of (177)Lu-Dotatate for Midgut Neuroendocrine Tumors. N Engl J Med.

[11] Kim SJ, Pak K, Koo PJ, Kwak JJ, Chang S (2015). The efficacy of (177)Lu-labelled peptide receptor radionuclide therapy in patients with neuroendocrine tumours: a meta-analysis. Eur J Nucl Med Mol Imaging.

[12] Hofman MS, Hicks RJ (2014). Peptide receptor radionuclide therapy for neuroendocrine tumours: standardized and randomized, or personalized?. Eur J Nucl Med Mol Imaging.

[13] Hubble D (2010). 177Lu-octreotate, alone or with radiosensitising chemotherapy, is safe in neuroendocrine tumour patients previously treated with high-activity 111In-octreotide. Eur J Nucl Med Mol Imaging.

[14] Kong G (2009). High-administered activity In-111 octreotide therapy with concomitant radiosensitizing 5FU chemotherapy for treatment of neuroendocrine tumors: preliminary experience. Cancer biotherapy & radiopharmaceuticals.

[15] van Essen M (2008). Report on short-term side effects of treatments with 177Lu-octreotate in combination with capecitabine in seven patients with gastroenteropancreatic neuroendocrine tumours. Eur J Nucl Med Mol Imaging.

[16] Claringbold PG, Brayshaw PA, Price RA, Turner JH (2011). Phase II study of radiopeptide 177Lu-octreotate and capecitabine therapy of progressive disseminated neuroendocrine tumours. Eur J Nucl Med Mol Imaging.

[17] Claringbold PG, Price RA, Turner JH (2012). Phase I-II study of radiopeptide 177Lu-octreotate in combination with capecitabine and temozolomide in advanced low-grade neuroendocrine tumors. Cancer biotherapy & radiopharmaceuticals.

[18] Yordanova A (2019). Peptide Receptor Radionuclide Therapy Combined With Chemotherapy in Patients With Neuroendocrine Tumors. Clinical nuclear medicine.

[19] Kong G (2014). Assessment of predictors of response and long-term survival of patients with neuroendocrine tumour treated with peptide receptor chemoradionuclide therapy (PRCRT). Eur J Nucl Med Mol Imaging.

[20] Kashyap R (2014). Favourable outcomes of Lu-octreotate peptide receptor chemoradionuclide therapy in patients with FDG-avid neuroendocrine tumours. Eur J Nucl Med Mol Imaging.

[21] De Vos M, Schreiber V, Dantzer F (2012). The diverse roles and clinical relevance of PARPs in DNA damage repair: current state of the art. Biochemical pharmacology.

[22] Scott CL, Swisher EM, Kaufmann SH (2015). Poly (ADP-ribose) polymerase inhibitors: recent advances and future development. J Clin Oncol.

[23] Donawho CK (2007). ABT-888, an orally active poly(ADP-ribose) polymerase inhibitor that potentiates DNA-damaging agents in preclinical tumor models. Clin Cancer Res.

[24] Liu X (2008). Potentiation of temozolomide cytotoxicity by poly(ADP)ribose polymerase inhibitor ABT-888 requires a conversion of single-stranded DNA damages to double-stranded DNA breaks. Molecular cancer research: MCR.

[25] Palma JP (2009). ABT-888 confers broad *in vivo* activity in combination with temozolomide in diverse tumors. Clin Cancer Res.

[26] Thomas HD (2007). Preclinical selection of a novel poly(ADP-ribose) polymerase inhibitor for clinical trial. Mol Cancer Ther.

[27] Chow JP (2013). PARP1 is overexpressed in nasopharyngeal carcinoma and its inhibition enhances radiotherapy. Mol Cancer Ther.

[28] Senra JM (2011). Inhibition of PARP-1 by olaparib (AZD2281) increases the radiosensitivity of a lung tumor xenograft. Mol Cancer Ther.

[29] McCluskey AG (2012). Inhibition of poly(ADP-Ribose) polymerase enhances the toxicity of 131I-metaiodobenzylguanidine/topotecan combination therapy to cells and xenografts that express the noradrenaline transporter. J Nucl Med.

[30] Al-Ejeh F (2013). Treatment of triple-negative breast cancer using anti-EGFR-directed radioimmunotherapy combined with radiosensitizing chemotherapy and PARP inhibitor. J Nucl Med.

[31] Nonnekens J (2016). Potentiation of Peptide Receptor Radionuclide Therapy by the PARP Inhibitor Olaparib. Theranostics.

[32] Purohit NK (2018). Potentiation of (177)Lu-octreotate peptide receptor radionuclide therapy of human neuroendocrine tumor cells by PARP inhibitor. Oncotarget.

[33] Jessop N, Hay R (1980). Characteristics of two rat pancreatic exocrine cell lines derived from transplantable tumors. In Vitro.

[34] Evers BM (1991). Establishment and characterization of a human carcinoid in nude mice and effect of various agents on tumor growth. Gastroenterology.

[35] Friedman HS (1988). Phenotypic and genotypic analysis of a human medulloblastoma cell line and transplantable xenograft (D341 Med) demonstrating amplification of c-myc. Am J Pathol.

[36] Allen M, Bjerke M, Edlund H, Nelander S, Westermark B (2016). Origin of the U87MG glioma cell line: Good news and bad news. Sci Transl Med.

[37] Biedler JL, Helson L, Spengler BA (1973). Morphology and growth, tumorigenicity, and cytogenetics of human neuroblastoma cells in continuous culture. Cancer Res.

[38] Biedler JL, Spengler BA (1976). Metaphase chromosome anomaly: association with drug resistance and cell-specific products. Science.

[39] Parry JJ (2007). Characterization of somatostatin receptor subtype 2 expression in stably transfected A-427 human cancer cells. Molecular imaging.

[40] Forssell-Aronsson E, Spetz J, Ahlman H (2013). Radionuclide therapy via SSTR: future aspects from experimental animal studies. Neuroendocrinology.

[41] Chamberlain CE (2018). A Patient-derived Xenograft Model of Pancreatic Neuroendocrine Tumors Identifies Sapanisertib as a Possible New Treatment for Everolimus-resistant Tumors. Mol Cancer Ther.

[42] Hofving T (2018). The neuroendocrine phenotype, genomic profile and therapeutic sensitivity of GEPNET cell lines. Endocrine-related cancer.

[43] Oddstig J, Bernhardt P, Nilsson O, Ahlman H, Forssell-Aronsson E (2006). Radiation-induced up-regulation of somatostatin receptor expression in small cell lung cancer *in vitro*. Nucl Med Biol.

[44] Taelman VF (2016). Upregulation of Key Molecules for Targeted Imaging and Therapy. J Nucl Med.

[45] Adant S, Shah GM, Beauregard JM (2019). Combination treatments to enhance peptide receptor radionuclide therapy of neuroendocrine tumours. Eur J Nucl Med Mol Imaging.

[46] Henderson, J. J. *et al*. Functional validation of the oncogenic cooperativity and targeting potential of tuberous sclerosis mutation in medulloblastoma using a MYC-amplified model cell line. *Pediatr Blood Cancer* **64**, 10.1002/pbc.26553 (2017). 10.1002/pbc.26553 28409891

[47] Duchemann B (2013). [Hypersensitivity to radiation therapy in a patient with tuberous sclerosis: biological considerations about a clinical case]. Cancer Radiother.

[48] Scarpa A (2019). The landscape of molecular alterations in pancreatic and small intestinal neuroendocrine tumours. Ann Endocrinol (Paris).

[49] Crumbaker, M., Emmett, L., Horvath, L. G. & Joshua, A. M. Exceptional Response to 177Lutetium Prostate-Specific Membrane Antigen in Prostate Cancer Harboring DNA Repair Defects. *JCO Precision Oncology*, 1–5, 10.1200/po.18.00237 (2019). 10.1200/PO.18.00237 35100671

[50] Lesueur P (2017). Poly-(ADP-ribose)-polymerase inhibitors as radiosensitizers: a systematic review of pre-clinical and clinical human studies. Oncotarget.

[51] Evers BM, Ishizuka J, Townsend CM, Thompson JC (1994). The human carcinoid cell line, BON. A model system for the study of carcinoid tumors. Annals of the New York Academy of Sciences.

[52] Kashyap R (2013). Rapid blood clearance and lack of long-term renal toxicity of (177)Lu-DOTATATE enables shortening of renoprotective amino acid infusion. Eur J Nucl Med Mol Imaging.

[53] Livak KJ, Schmittgen TD (2001). Analysis of relative gene expression data using real-time quantitative PCR and the 2(-Delta Delta C(T)) Method. Methods.

[54] Euhus DM, Hudd C, LaRegina MC, Johnson FE (1986). Tumor measurement in the nude mouse. J Surg Oncol.

[55] Clifton-Bligh RJ (2013). Improving diagnosis of tumor-induced osteomalacia with Gallium-68 DOTATATE PET/CT. J Clin Endocrinol Metab.

[56] Dorow DS (2006). Multi-tracer small animal PET imaging of the tumour response to the novel pan-Erb-B inhibitor CI-1033. Eur J Nucl Med Mol Imaging.

