# Supplementary material for: Enhancing the anti-tumour activity of 177Lu-DOTA-octreotate radionuclide therapy in somatostatin receptor-2 expressing tumour models by targeting PARP
Source: Sci Rep. 2020 Jun 23;10:10196. doi: 10.1038/s41598-020-67199-9 (PMC7311440; doi:10.1038/s41598-020-67199-9)
Supplement: Supplementary file 1 — Supplementary information. [file 41598_2020_67199_MOESM1_ESM.pdf]

Supplementary Information

Enhancing the anti-tumour activity of ^{177}Lu-DOTA-octreotate radionuclide therapy in somatostatin receptor-2 expressing tumour models by targeting PARP.

*Carleen Cullinane[a,c], *Kelly Waldeck[a,] Laura Kirby[a], Buck E Rogers[e], Peter Eu[b], Richard W Tothill[a,d] and Rodney J Hicks[a,b,c]

[a]Division of Cancer Research and [b]Department of Cancer Imaging, Peter MacCallum Cancer Centre, Melbourne, Victoria, Australia
[c]Sir Peter MacCallum Department of Oncology and [d]Department of Clinical Pathology and Centre for Cancer Research, University of Melbourne, Parkville, Victoria, Australia,
[e]Department of Radiation Oncology, Washington University School of Medicine, St Louis, MO, 63110, USA

Corresponding Author: Carleen Cullinane
Email: carleen.cullinane@petermac.org
Phone: +61-3-85597120

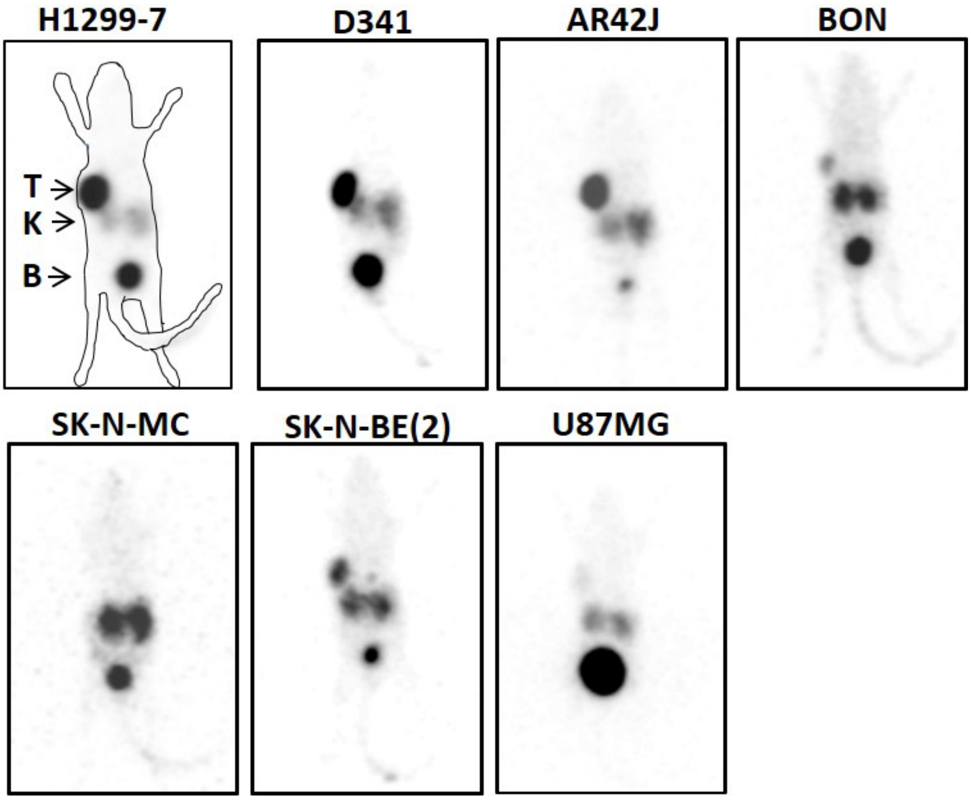

Supplementary Figure S1: ^{68}GaTate PET imaging of tumour moels *in vivo*. Tumour bearing mice were imaged on a small animal PET scanner at 1 hr post injection with ^{68}GaTate. Representative maximum intensity projection images are shown for each tumour model. T, tumour; K, kidney; B, bladder.
